# MHC Class II-Restricted Epitopes Containing an Oxidoreductase Activity Prompt CD4^+^ T Cells with Apoptosis-Inducing Properties

**DOI:** 10.3389/fimmu.2015.00449

**Published:** 2015-09-02

**Authors:** Elin Malek Abrahimians, Vincent A. Carlier, Luc Vander Elst, Jean-Marie R. Saint-Remy

**Affiliations:** ^1^Center for Molecular and Vascular Biology, University of Leuven, Leuven, Belgium; ^2^ImCyse SA, Leuven, Belgium

**Keywords:** MHC class II epitopes, oxidoreductase activity, cytolytic CD4^+^ T cells, apoptosis, immune suppression

## Abstract

Abrogating an unwanted immune response toward a specific antigen without compromising the entire immune system is a hoped-for goal in immunotherapy. Instead of manipulating dendritic cells and suppressive regulatory T cells, depleting effector T cells or blocking their co-stimulatory pathways, we describe a method to specifically inhibit the presentation of an antigen eliciting an unwanted immune reaction. Inclusion of an oxidoreductase motif within the flanking residues of MHC class II epitopes polarizes CD4^+^ T cells to cytolytic cells capable of inducing apoptosis in antigen presenting cells (APCs) displaying cognate peptides through MHC class II molecules. This novel function results from an increased synapse formation between both cells. Moreover, these cells eliminate by apoptosis bystander CD4^+^ T cells activated at the surface of the APC. We hypothesize that they would thereby block the recruitment of cells of alternative specificity for the same autoantigen or cells specific for another antigen associated with the pathology, providing a system by which response against multiple antigens linked with the same disease can be suppressed. These findings open the way toward a novel form of antigen-specific immunosuppression.

## Introduction: A Distinct Subset of CD4^+^ T Cells

CD4^+^ T cells with cytotoxic activity have been occasionally described over the past 30 years (1–3). Their role in infection has been recognized, both during natural disease (4–6) and as an outcome of immunization (7, 8). Moreover, their antitumor potential seems to have been underestimated (9). Despite recent advances characterizing these cells as end-stage differentiated cells (10) and describing their cytolytic function through Fas–Fas ligand interaction and perforin/granzymes activity (11), the conditions under which they can be elicited remain poorly understood and their physiological relevance has not been fully established (12, 13).

CD4^+^ T cells can also differentiate into regulatory T cells (Tregs). Natural Tregs are selected in the thymus and show specificity for auto as well as alloantigens (14). The identification of Foxp3 as a master transcription regulator and subsequent gene profiling (15) have established their identity, with a phenotype, including constitutive expression of CD25, GITR, ICOS, CTLA-4, and CD103, production of IL-10, and absence of CD127 (16). Natural Tregs are essential for the control of autoimmunity and have shown a therapeutic potential in experimental autoimmune diseases and allograft rejection (17), but their use is still limited by difficulties of expanding stable populations, their lack of antigen specificity and concern of non-specific effect related to the production of suppressive cytokines (18).

Over recent years, a second category of Tregs has been described, called adaptive or induced Tregs (iTregs). These cells can be obtained both *in vitro* in absence of antigen by incubation with IL-10, vitamin D3, dexamethasone, or IFN-α (Tr1) and *in vivo* by antigen administration via the oral route (Th3) or by peptide administration in the absence of adjuvant via the transmucosal route (19) or subcutaneously (20). The molecular mechanisms underlying the function of iTregs are poorly understood, reflecting their heterogeneity. They do not express Foxp3, except for transient expression in the presence of TGF-β (21). Some iTregs overexpress T-bet when elicited by peptide administration without adjuvant (19), or GATA-3 after intranasal administration of ovalbumin (22), suggesting that the phenotype of iTregs and their functional properties vary according to initial T cell lineage commitment and degree of maturation (23). iTregs share an anergic status and limited capacities for *in vitro* expansion (18).

We describe here what we consider as a *bona fide* new and distinct functional subset of CD4^+^ T cells. The uniqueness of these cells are based on two key observations: one, these cells can be generated from any of the major subsets tested so far, including highly polarized Th1, Th2, and Th17 cells; two, in parallel to acquisition of apoptosis-inducing properties, such cells acquire a phenotype of terminally differentiated effector memory cells.

## Induction of Cytolytic CD4^+^ T Cells

Formation of a synapse in between an antigen presenting cell (APC) expressing a MHC class II molecule presenting an antigen-derived epitope and a CD4^+^ T cell constitutes the earliest step of recognition by the adaptive immune system, and as such represents an ideal target for intervention.

The crystal structure of a MHC class II molecule shows that the cleft in which an epitope can be accommodated remains open on both sides, providing the opportunity to bind epitopes exceeding the sequence bound to the cleft. Thus, epitopes up to 20 amino acids have been described as being presented by class II molecules, although the sequence recognized by the TCR is limited to 8–9 amino acids. Retrospectively, it is surprising to realize that this general observation has attracted very limited attention. Residues located outside of the cleft, the so-called flanking residues, have been considered almost exclusively to identify their role in strengthening either the binding of the epitope in the cleft or alternatively the strength of TCR binding. Indeed, the nature of the amino acids just adjacent to positions P1 and P9, which correspond to the first and the last anchoring residues for the epitope, has been shown to influence such binding (24). This is all the more surprising as the strength of the synapse in between an APC and a CD4^+^ T cells is known to be one of the factors deciding upon the fate of the CD4^+^ T cells (25). Recent experiments have, however, demonstrated that modifications in flanking residues of a variety of epitopes can affect TCR recognition and CD4^+^ T cell function (26, 27).

Earlier research on one of the major class II-restricted T cell epitopes (p21–35) of the Der p 2 allergen derived from the house dust mite, *Dermatophagoides pteronyssinus*, showed that this peptide elicited CD4^+^ T cells, which appeared to eliminate APCs in culture (28, 29). The determinants in this peptide sequence that lead to this remarkable property were unknown. Extensive investigation of this epitope unveiled the presence of an intact amino terminal oxidoreductase motif (CxxS motif, where C stands for cysteine, S for serine, and x for any other amino acid) located within the flanking residues. This motif is characteristic of monocysteinic glutaredoxins (30), which are known to exert a nucleophilic attack on disulfide bridges and to create as such stable intermediates (31).

Amino acid substitution assays demonstrated that the presence of this motif in the Der p 2 (p21–35) sequence is essential for acquisition of cytolytic activities toward APCs (32). Introducing such motif in the flanking residues of other T cell epitopes demonstrates that the cytolytic properties are not limited to the specific Der p 2 epitope sequence context. The natural CxxS motif was further optimized by introducing a second cysteine, making a CxxC motif (33) presenting a higher oxidoreductase and reinforcing the cytolytic properties. This novel function results from an increased synapse formation between the APC and the antigen-specific CD4^+^ T cells. Carlier et al. further demonstrated that the target for the reduction was a constrained disulfide bridge located in the second extracellular domain of the CD4 molecule itself.

## Strength of Synapse Formation and Acquisition of a Cytolytic Phenotype

The strength of the synapse is a determining factor for the fate of CD4^+^ T cells, low strength leads to ignorance or induction of anergy, while excessive strength could prime activation-induced cell death. The CD4 molecule binds via its first extracellular domain to residues located in the MHC class II molecule. By doing so, the synapse itself, which is by nature of low affinity, is physiologically strengthened. Reducing the disulfide bridge allows the formation of homodimers or polymers of CD4, thereby further strengthening the synapse, as demonstrated by increased number of doublets in between APCs and CD4^+^ T cells (32).

CD4 contains an intracellular domain with subdomains for the recruitment of kinases associated with early signaling. Exposure of naïve CD4^+^ T cells to epitopes with a CxxC motif located in flanking residues results in increased rates of recruitment of Lck and subsequently ZAP70, forming an activation complex with CD3. An increase in the kinetics and extent of CD3ζ phosphorylation and degradation further indicated that the increase in the activation followed a physiological pathway. Furthermore, we observed that the increased rate of this early signaling complex was followed by sustained activation of PI3K and AKT. Phosphorylation of AKT is a crucial event for cells, as it drives the proliferation capacity of the cells, their metabolism, and phenotype adoption. Thus, increased phosphorylation of AKT resulted in prevention of nuclear migration of FOXO-3a, triggered the aerobic glycolytic pathway shared by effector cells and activated the mTOR complex pathway (our unpublished data). Of much interest, these activation and metabolic properties are opposite to the ones observed with Tregs, in which AKT activation is reduced and the metabolism switched to oxidated lipid phosphorylation, indicating already that on phenotypical grounds, cells exposed to epitopes containing the CxxC motif adopted a diametrically opposite phenotype as Tregs do.

Functionally speaking, cells exposed to epitopes containing a CxxC motif promptly adopted a phenotype of effector memory cells, characterized by rapid loss of expression of CD62L and high expression of CD44. Other relevant surface markers include CD25, a characteristic shared with Tregs, and which provides cells with the possibility of utilizing IL-2 when present at low concentrations leading to a proliferative advantage over alternative effector cells. Low expression of CD28 and increased expression of CTLA-4 are also shared features with Tregs. At the transcriptional level, cells are characterized by co-expression of T-bet and GATA-3, considered as being exclusive from each other in effector cells, Th1 expressing T-bet and Th2 cells expressing GATA-3. Worth mentioning is that, under no circumstances, it was possible to detect Foxp3, at protein or even mRNA level (Malek Abrahimians et al., in preparation). The production of cytokines is essentially limited to IFN-γ. FasL and granzyme B are transcribed and the proteins produced. Both actively participate in the induction of apoptosis of target cells, such as APCs with which a synapse is formed. Perforin can be transcribed but seemingly with no production of the protein. Blocking experiments have confirmed the direct participation of both granzyme B and FasL in the cytolytic activity of the cells, while inhibitors of perforin showed no effect (32).

## Conversion of Polarized Cells and Induction of Bystander CD4^+^ T Cell Apoptosis

We observed that polarized cells exposed to their cognate epitope in the presence of a CxxC motif progressively lose their phenotypic characteristics to acquire cytolytic properties. Interestingly enough, once the cytolytic properties are acquired, cells seemingly do not revert to their initial phenotype even under highly polarizing conditions (Carlier et al., in preparation).

The question was extended to bystander CD4^+^ T cells to determine whether the technology would be effective in a multi-epitope and multi-antigen pathology; in other words, whether it was possible to suppress a polyclonal CD4^+^ T cell response using a single epitope from a single antigen. We demonstrated that CD4^+^ T cells that had acquired cytolytic potential, were capable of eliminating by apoptosis whatever CD4^+^ T cells, provided these cells were activated at the surface of the same APC. This apoptosis occurred independently of the elimination of the APC itself (32).

Taken altogether, the combination of the possibility to convert naïve or polarized cells into a cytolytic phenotype, with the possibility to eliminate by apoptosis activated bystander CD4^+^ T cells, constitutes a comprehensive approach with potential for therapeutic intervention.

## Pre-Clinical Evidence toward a Therapeutic Application

In a model of experimental allergic asthma, as induced by nasal instillation of recombinant allergens prepared from the house dust mite, *D. pteronyssinus*, immunization with a peptide encompassing a class II-restricted epitope of either Der p 1 or Der p 2 was sufficient to eliminate the bronchial reactivity to exposure not only to the allergen serving to induce asthma but also to an alternative allergen. Besides, and more importantly, such a treatment eliminated the bronchial reactivity to a non-specific stimulus largely used in a clinical setting, namely acetylcholine, demonstrating that such an approach is able to reduce both specific and non-specific bronchial reactivity.

In a model of skin graft rejection, pre-immunization of female C57BL/6 mice with a single epitope derived from a single antigen encoded by the Y chromosome was sufficient to obtain full tolerance to the grafting of a male skin. This model also established that mice having rejected a graft, and therefore under condition of higher reactivity to alloantigens were rendered tolerant to a subsequent graft if pretreated with the epitope of the Dby antigen. Additionally, mice pre-immunized and having tolerated a first graft were tolerant to a second identical graft 4 months later and with no intermediate treatment, providing an indication of the *in vivo* persistence of such Dby-specific cytolytic CD4^+^ T cells.

In a model of multiple sclerosis, either pre-immunization with a class II-restricted epitope from the MOG protein (a constituent of the myelin gain) or immunization initiated weeks after disease induction, it was possible to prevent the appearance of clinical signs or, to some extent, reduce such signs. Strikingly, in such a model, gains of myelin were reconstituted in a context in which it could be observed that the inflammatory infiltrates in the central nervous system white matter or the spinal cord had been completely eliminated. Interestingly, infusion of cells, in the same model, converted *in vitro* to a cytolytic phenotype was efficient to either prevent or suppress the disease. Noticeably, a clinical trial with patients with relapsing–remitting multiple sclerosis has been recently initiated using the same principle.

In a model of gene therapy, in which the immune response toward the viral vector prohibits an effective administration and expression of a transgene, we have demonstrated that administration of a peptide encompassing a class II-restricted epitope of hexon 6, a capsid protein of adenovirus 5, was sufficient to prevent such an immune response, allowing re-administration of the same construct, use of a significantly lower dose of construct, use of improved transgenes, and prolongation of transgene expression. Considering that, in terms of immunogenicity, adenovirus represents the worst-case scenario, we surmise that alternative viruses, such as the adeno-associated virus (AAV), would be amenable to the same approach. Importantly, the same method could be used for gene vaccination.

In the spontaneous non-obese diabetes (NOD) mouse model of type 1 diabetes, immunization with a MHC class II-restricted epitope derived from glutamic acid decarboxylase (GAD65), a major autoantigen, prevents autoimmune destruction of pancreatic β-cells. Furthermore, passive transfer of *in vitro* derived cytolytic CD4^+^ T cells also prevented diabetes. Interestingly, this setting confirms that immunization with a single epitope is sufficient to prevent autoimmunity toward multiple antigens implicated in the disease process.

## Risk Assessment

There is a theoretical risk of reversibility or conversion of cytolytic CD4^+^ T cells to pathogenic effector cells. However, efforts to force this under stringent *in vitro* conditions have been unsuccessful (our unpublished data). Furthermore, the number of generated cytolytic cells is very limited as compared to bulk of pathogenic effector cells present in the setting of an ongoing disease, indicating that, even in case of reversibility toward an effector phenotype, the clinical relevance of this would not be perceptible.

Although the concept makes use of a single epitope selected from an antigen directly implicated in the disease under investigation, the very fact that cytolytic CD4^+^ T cells eliminate activated bystander CD4^+^ T cells could, in theory, lead to elimination of useful effector cells toward, for instance, a microorganism. However, as the activation and cytolytic properties of the cells requires the formation of a synapse with a cognate epitope, we have to envision a situation in which all the immune response toward the microorganism is elaborated at the same time and in the same location as the response that has to be eliminated. In a large majority of the case, and in particular in organ-specific autoimmune diseases, the pathogenic response is elaborated in the draining regional lymph nodes, which is not likely to be the port of entry of the microorganism. We therefore deem that the likelihood of increasing the susceptibility to a concomitant infection is remote.

Any intervention modifying the fate of a cell carries the risk of increasing tumorigenic potential. However, to evaluate this risk, we have analyzed the caryotype of these transformed cells and have not identified any significant alterations in comparison to effector cells under recurrent stimulation. Moreover, measurement of the telomerase activity to evaluate the status of senescence of such cells has not showed reduced activity (our unpublished data).

## Oxidoreductase-Containing Versus Unmodified Epitopes

Peptides encompassing class II-restricted epitopes have been used in several settings in an attempt to control disease processes. Such peptides are administered via different routes, often transmucosal, and in absence of adjuvant. Some results have been observed in animal models, yet very little if any in the clinic (34).

The mechanism of action of such unmodified epitopes is related to restimulation-induced cell death (RICD) (35). This mechanism is linked to the extrinsic pathway of apoptosis induction, a mechanism associated with signaling through the TNFR superfamily, including Fas–FasL interaction and the formation of DISC, the death-inducing signaling complex, recruiting and activating caspases 8 and 10. This is opposed to the intrinsic pathway in which depolarization of the mitochondrial membrane activates caspase 9. RICD prevents uncontrolled expansion of an immune response and is responsible for the contraction phase following an immune response, in which the number of activated CD4^+^ T cells drop dramatically, to maintain a small population entering into a cycle of memory phenotype conversion.

The results obtained in animal experiments using unmodified epitopes administered without adjuvant is deemed to depend on the elimination, by the induction of apoptosis by the extrinsic pathway, of the CD4^+^ T lymphocytes specific only for the very antigen from which the epitope has been selected. This explains the significant, though partial beneficial effect of such approach. However, in a clinical setting, wherein a large number of antigens are involved, the elimination of a single CD4^+^ T cell population will provide very limited, if any benefit, very much in keeping with the absence of clinical efficacy (36).

In the present setting, instead of reducing the size of a single CD4^+^ T cell population specific for an epitope of a single antigen, the intrinsic pathway of apoptosis induction is used in combination with the extrinsic pathway. Effector cells utilizing both the intrinsic and extrinsic apoptosis induction pathways provide an active method to eliminate effector CD4^+^ T cells of alternative specificity (alternative epitopes of the same antigen and alternative antigens) (37), i.e., eliminating a much larger spectrum of effector cells associated with a disease process than unmodified epitopes. Besides, elimination of the APCs with which the synapse is formed provides a means to prevent the recruitment of new cells in the pathological process. These fundamental differences in between the mode of action of cytolytic CD4^+^ T cells and the mere depletion of single effector cell population establish the superiority of the approach.

## Concluding Remarks

These pre-clinical observations on oxidoreductase-containing epitopes and their elicitation of CD4^+^ T cells capable of specifically eliminating APCs presenting the nominal peptide opens a door to a novel therapeutic strategy in immune disease and leads to hypothesize that any class II-restricted T cell epitope can be modified by addition of a oxidoreductase motif in order to induce specific CD4^+^ T cells with apoptosis-inducing properties and thus overcome any unwanted class II-restricted immune response. The high homology in between the synapse created at MHC class II level in mouse and in human leads to the assumption that the observations carried out in pre-clinical models could be extrapolated to human cells. Indeed, we have demonstrated efficacy in converting *in vitro* human CD4^+^ T cells to a cytolytic phenotype, allowing application to human disease in the near future.

## Conflict of Interest Statement

This research was conducted in the absence of any commercial or financial relationships that could be construed as a potential conflict of interest.
